# Degradation of key photosynthetic genes in the critically endangered semi-aquatic flowering plant *Saniculiphyllum guangxiense* (Saxifragaceae)

**DOI:** 10.1186/s12870-020-02533-x

**Published:** 2020-07-08

**Authors:** Ryan A. Folk, Neeka Sewnath, Chun-Lei Xiang, Brandon T. Sinn, Robert P. Guralnick

**Affiliations:** 1 Department of Biological Sciences, Mississippi State University, Mississippi, Mississippi State USA; 2 Florida Museum of Natural History, University of Florida, Gainesville, Florida USA; 3 CAS Key Laboratory for Plant Diversity and Biogeography of East Asia, Kunming Institute of Botany, Chinese Academy of Sciences, Kunming, Yunnan P. R. China; 4 Department of Biology & Earth Science, Otterbein University, Westerville, OH USA

**Keywords:** Plastid genome, Plastome, Pseudogene, Organelle, Saxifragaceae, *Saniculiphyllum*

## Abstract

**Background:**

Plastid gene loss and pseudogenization has been widely documented in parasitic and mycoheterotrophic plants, which have relaxed selective constraints on photosynthetic function. More enigmatic are sporadic reports of pseudogenization and loss of important photosynthesis genes in lineages thought to be fully photosynthetic. Here we report the complete plastid genome of *Saniculiphyllum guangxiense*, a critically endangered and phylogenetically isolated plant lineage, along with genomic evidence of reduced chloroplast function. We also report 22 additional plastid genomes representing the diversity of its containing clade Saxifragales, characterizing gene content and placing variation in a broader phylogenetic context.

**Results:**

We find that the plastid genome of *Saniculiphyllum* has experienced pseudogenization of five genes of the *ndh* complex (*ndhA*, *ndhB, ndhD*, *ndhF*, and *ndhK*), previously reported in flowering plants with an aquatic habit, as well as the surprising pseudogenization of two genes more central to photosynthesis (*ccsA* and *cemA*), contrasting with strong phylogenetic conservatism of plastid gene content in all other sampled Saxifragales. These genes participate in photooxidative protection, cytochrome synthesis, and carbon uptake. Nuclear paralogs exist for all seven plastid pseudogenes, yet these are also unlikely to be functional.

**Conclusions:**

*Saniculiphyllum* appears to represent the greatest degree of plastid gene loss observed to date in any fully photosynthetic lineage, perhaps related to its extreme habitat specialization, yet plastid genome length, structure, and substitution rate are within the variation previously reported for photosynthetic plants. These results highlight the increasingly appreciated dynamism of plastid genomes, otherwise highly conserved across a billion years of green plant evolution, in plants with highly specialized life history traits.

## Background

Plastid genome structure and content is highly conserved among most of the ~ 500,000 species of land plants and their closest green algal relatives [1]. Widespread loss or pseudogenization of photosynthetic genes is a familiar feature of the plastids of diverse non-photosynthetic plant lineages, reflecting the reduced need for photosynthetic genes in lineages with heterotrophic strategies [2]. Accumulating evidence, however, has increasingly documented the pseudogenization and loss of “accessory” photosynthetic genes, only conditionally essential under stress, in fully photosynthetic plants. Although not universal, many of these pseudogenizations and losses are associated with highly specialized life history traits such as aquatic habit [3–5], carnivory [6, 7], and a mycoheterotrophic life-stage [1]; the functional significance of these losses remains enigmatic [8].

Plastid gene loss and pseudogenization is currently best studied in mycoheterotrophic, primarily non-photosynthetic plants, which are under relatively relaxed selection on photosynthetic function. Remarkably, these plant lineages tend to follow a relatively predictable gene loss sequence, with early loss of photosynthetic accessory genes, primarily restricted to the *ndh* complex [2], which is involved in photooxidative protection under stressful conditions [8, 9]. This is eventually followed, in taxa with a longer evolutionary history of mycoheterotrophy, by loss of core photosynthetic genes. Housekeeping genes unrelated to photosynthesis, such as plastid ribosomal genes, are very resistant to loss [2]. Plastid gene content is less well-studied in fully photosynthetic life histories, but examples so far are consistent with this model. Gene loss to date has been restricted to portions of the *ndh* complex [3, 8, 10–12], which appears to be non-essential in model systems in the absence of abiotic stress [9, 13]. Whether such losses of a functional copy from the plastid genome truly represent a loss of function remains uncertain, as there are many examples of gene loss or pseudogenization in organellar genomes accompanied by functional transfer to the nuclear genome [14, 15].

*Saniculiphyllum guangxiense* C.Y. Wu & T.C. Ku is a semi-aquatic flowering plant now restricted to a miniscule area in Yunnan province, China. It grows partially submersed in the flow of small shaded waterfalls, and is critically endangered, with only four small extant populations in an area ~ 10 km^2^ known to science, as well as several other populations known to have been extirpated within the last 30 years [16]. Consistent with the isolated morphological and ecological traits of this lineage within the family Saxifragaceae, its phylogenetic affinities remain uncertain. The most recent attempts to place this species [16–18] exhibit strong disagreement. Xiang et al. [16], using six loci generated by Sanger sequencing, could not confidently place this lineage beyond its membership in the Heucheroid clade, while [17], using the same genetic loci, were able to place this lineage with 0.93–1.0 posterior probability (depending on the analysis) as sister to the *Boykinia* group, a difference Deng et al. [17] attribute to alignment differences in a single rapidly evolving genetic locus (ITS). Relationships in these studies based on Sanger sequencing data differ substantially in several areas from those recovered on the basis of more than 300 nuclear genes [18], where *Saniculiphyllum* was placed with moderate bootstrap support (80%) as sister to a clade containing the *Astilbe* and *Boykinia* groups.

In the course of organellar genome surveys across Saxifragales, we found anomalous photosynthetic gene sequences in *Saniculiphyllum.* Here, we report new plastid genome sequences of phylogenetically pivotal taxa, analyze plastid gene evolution across the Saxifragales, and place the *Saniculiphyllum* plastid genome in a phylogenetic context to assess evolutionary relationships and rates of plastid evolution. We seek to test whether *Saniculiphyllum* (1) has evidence for reduced photosynthetic function via pseudogenization of accessory photosynthetic genes, and (2) whether there is evidence for restored function of plastid pseudogenes via nuclear paralogs.

## Results

### Assembly results

For all samples, we successfully assembled a complete circular genome using NOVOPlasty. We individually confirmed all sequence features noted below by mapping the reads back to the assembly and found no evidence of misassembly.

### Basic genome features

**Fig. 1:**
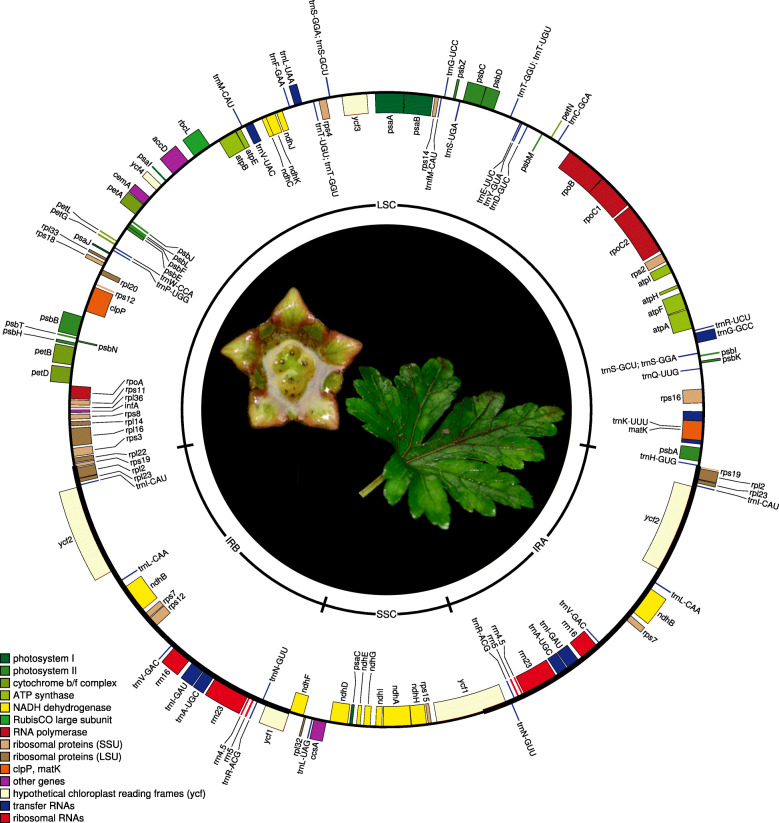
Gene map of the *Saniculiphyllum* plastome built using OrganellarGenomeDRAW [19]; genes marked on the outside face of the circle are transcribed counter-clockwise and those inside the circle are transcribed clockwise. Center photo: *Saniculiphyllum* flower and leaf; photo credit: C.-L. X

*Saniculiphyllum* has a chloroplast genome 151,704 bp long (Fig. 1). The large-scale structure of the genome is canonical for land plants, with an inverted repeat (26,109 bp) separating the large-single-copy region (LSC; 84,479 bp) and small-single-copy region (SSC, 15,007 bp). Excluding putative pseudogenes (below), gene content was as expected [20], comprising 73 distinct protein-coding genes, 30 tRNA genes, and 4 rRNA genes.

### Evidence for pseudogenization

We used the presence of premature stop codons, frame shift mutations, missing canonical stop codons, and very large deletions as our criteria for pseudogenization (reviewed in [21]). We used gene models in the previously published *H. parviflora* var. *saurensis* genome [22] as well as comparisons across the newly annotated Saxifragales genomes presented here. We found genomic evidence for pseudogenization in 5 genes of the *ndh* complex (*ndhA, ndhB, ndhD, ndhF*, and *ndhK*), and two other photosynthetic genes (*cemA, ccsA*), summarized in Table S2, Additional file 1). These were either driven by frame-shift mutations (*ccsA, ndhA, ndhD*, and *ndhF*) or by premature stop codons without a frameshift (due to a point mutation in *ndhB* and a short inversion in *ndhK*). Three genes (*cemA*, *ndhD,* and *ndhF*) lack much of the conserved gene sequence due to large deletions > 100 bp. Among these, *cemA* has no premature stop codons, but it has an unconventional predicted protein size (5 extra amino acids) in a gene that otherwise shows no size variation in Saxifragales; while lacking 18% of the 3′ end of this gene, the *Saniculiphyllum* copy has 137 additional bp before a novel stop codon, the sequence of which is homologous with adjacent intergenic spacers in its relatives, making it unlikely that this sequence is functional. Additionally, frameshift has resulted in the loss of the conserved stop codon site of *ndhA*. Predicted proteins of the three genes with large deletions (*cemA*, *ndhD,* and *ndhF*) have hydrophobicity outside the range of variation of other Saxifragales (*cemA* 50% hydrophobic amino acids vs. the 95% confidence interval for other Saxifragales [50.4, 52.2%]; *ndhD* 47.8% vs. [62.2, 63.6%]; *ndhF* 54.9% vs. [55.6, 58.2%]).

### Evidence for paralogs of pseudogenes

For the three genes with large deletions (*cemA*, *ndhD,* and *ndhF*), we used the *Leptarrhena* sequence for the missing DNA to probe for potential nuclear or mitochondrial paralogs that could be functional; otherwise we used the entire CDS of this taxon. For all seven novel pseudogenes, we found evidence of paralogs outside of the assembled chloroplast genome, some of which are more conserved in sequence and lack the anomalous features of plastid pseudogenes (Supplementary Figs. S1–7). This includes copies of *cemA*, *ndhD,* and *ndhF* without the large deletions found in the plastid copy. However, with the exception of partial assembled sequences of *ndhF,* these paralogs all have either the same premature stop codons of the plastid copy or novel premature stop codons and are also unlikely to be functional. These paralogs likely originate in the nucleus on the basis of sequence coverage, which was orders of magnitude lower (SPAdes calculated *k*-mer coverage ~ 1-5X) than that expected for either the plastid or the mitochondrion (*k*-mer coverage 100-2000X).

With the exception of *ndhK,* where we recovered 4 independent lineages of *Saniculiphyllum* paralogs, gene genealogies (Figs. S1–7, Additional file 1) were consistent with a recent origin of paralogs of the seven pseudogenes. In the *ccsA* gene genealogy, the *Saxifraga stolonifera* Curtis plastid ortholog was placed within a *Saniculiphyllum* clade without support, but otherwise (*cemA, ndhA, ndhB, ndhD, ndhF*) the *Saniculiphyllum* paralogs were recovered as monophyletic.

### Other anomalous features

Several genes show slight variations in within-frame start and stop codon positions in Saxifragales, but *Saniculiphyllum* shows more variation than any other species we sampled, with four genes showing unique CDS terminations (*atpB, cemA, rpl20, ycf2*; Table S2, Additional file 1), of which none but *rpl20* show any size variation in other Saxifragales species. While still within the typical length of photosynthetic plastid genomes, the *Saniculiphyllum* plastid genome as a whole was significantly smaller than the mean for Saxifragales species (one-tailed t-test, *p* = 1.485e-10).

We indirectly measured plastid genome copy number relative to background (mostly nuclear) DNA by calculating the percent of total genomic DNA mapping to the plastid genome. Lower values suggest lower copy number, either through fewer plastids or fewer genome copies per plastid. Interestingly, this was significantly smaller in *Saniculiphyllum* (3.4%) compared to other Saxifragales (one-tailed t-test, *p* = 1.629e-07); the mean of our Saxifragales species sampled here was 10.1%, identical to a mean of 10.1% recovered with further Saxifragaceae species sampled in [22].

### Plastid genome diversity in Saxifragales

The new plastid genomes sequenced in this study have no evidence of any structural rearrangements, underlining the strong conservatism of plastid genome structure in Saxifragales [20, 23] (the rare exception is Haloragaceae, distantly related to taxa discussed here; see [23, 24]). Other than *Saniculiphyllum*, we found no evidence at the DNA sequence level for pseudogenes beyond those previously documented for Saxifragales [20, 23] and many other angiosperms, namely *ycf15* and pseudogenes created by IR (inverted repeat) region boundaries within *ycf1* and *ycf2* (cf. Table S2, Additional file 1). The IR region, a major contributor to plastid genome size variation [25], shows similar trends (Table 1) to those documented previously in Saxifragales, where much like genome structure it is highly conserved [20]. However, the family Saxifragaceae shows a trend towards reduction, with some of the smallest IR regions in Saxifragales.

**Table 1:** Summary of new chloroplast genome sequences reported in this paper

Species	Field collecting location	Sequencing technology	Collection data (Herbarium)	Genbank accession	Total sequencing effort (millions of reads)	Plastid genome mean coverage (reduced data)	Plastid assembly length	Inverted repeat length
*Boykinia aconitifolia*	Alleghany County, North Carolina, U.S.A.	BGI-SEQ	Folk 249 (FLAS)	MN496058	734.3	374.8×	154,368 bp	25,769 bp
*Cercidiphyllum japonicum*	Cultivation	BGI-SEQ	Whitten 5886 (FLAS)	MN496059	799.1	394.8×	159,897 bp	26,434 bp
*Daphniphyllum macropodum*	Cultivation	BGI-SEQ	Whitten 5884 (FLAS)	MN496060	684.5	66.0×	160,408 bp	26,605 bp
*Fortunearia sinensis*	Cultivation	BGI-SEQ	Folk 253 (FLAS)	MN496061	727.7	90.2×	159,413 bp	26,274 bp
*Heuchera abramsii*	Los Angeles County, California, U.S.A.	Illumina HiSeq	Folk I-40 (OS)	MN496062	211.8	710.3×	155,527 bp	25,838 bp
*Heuchera alba*	Pendleton County, West Virginia, U.S.A.	Illumina HiSeq	Folk 63 (OS)	MN496063	160.8	252.4×	155,360 bp	25,632 bp
*Heuchera caespitosa*	Ventura County, California, U.S.A.	Illumina HiSeq	Folk 48 (OS)	MN496064	171.7	490.5×	155,520 bp	25,834 bp
*Heuchera eastwoodiae*	Yavapai County, Arizona, U.S.A.	Illumina HiSeq	Folk 35 (OS)	MN496065	82.3	336.0×	155,174 bp	25,605 bp
*Heuchera grossulariifolia var. grossulariifolia*	Idaho County, Idaho, U.S.A.	Illumina HiSeq	Folk 160 (OS)	MN496066	226.6	742.7×	155,493 bp	25,645 bp
*Heuchera longipetala* var. *longipetala*	Cultivation	Illumina HiSeq	Folk I-21 (OS)	MN496067	223.6	768.3×	155,418 bp	25,638 bp
*Heuchera mexicana* var. *mexicana*	Cultivation	BGI-SEQ	Folk I-51 (OS)	MN496068	710.8	150.3×	155,451 bp	25,638 bp
*Heuchera parvifolia var. utahensis*	Cultivation	Illumina HiSeq	Folk I-56 (OS)	MN496069	273.3	738.5×	155,350 bp	25,633 bp
*Leptarrhena pyrolifolia*	Skagit County, Washington, U.S.A.	BGI-SEQ	J.V. Freudenstein 3069 (FLAS)	MN496070	752.2	253.8×	155,055 bp	25,781 bp
*Mitella diphylla*	Monroe County, Ohio, U.S.A.	Illumina HiSeq	Folk 88 (OS)	MN496071	230.8	357.0×	155,646 bp	25,753 bp
*Mitella pentandra*	King County, Washington, U.S.A.	Illumina HiSeq	Folk 128 (OS)	MN496072	264.4	341.2×	155,470 bp	25,620 bp
*Mukdenia rossii*	Cultivation	BGI-SEQ	Folk 259 (FLAS)	MN496073	799.0	330.9×	156,927 bp	25,585 bp
*Oresitrophe rupifraga*	Cultivation	BGI-SEQ	Folk 257 (FLAS)	MN496074	678.1	261.3×	156,787 bp	25,586 bp
*Ribes nevadense*	Shasta County, California, U.S.A.	BGI-SEQ	Nelson 2018–028 (FLAS)	MN496075	782.7	234.3×	157,715 bp	25,887 bp
*Ribes roezlii*	Shasta County, California, U.S.A.	BGI-SEQ	Nelson 2018–027 (FLAS)	MN496076	723.6	262.7×	157,781 bp	25,967 bp
*Rodgersia sambucifolia*	Cultivation	BGI-SEQ	R.A. Folk 266 (FLAS)	MN496077	637.3	193.2×	157,289 bp	25,556 bp
*Saniculiphyllum guangxiense*	Funing County, Yunnan, China	Illumina HiSeq	Xiang 1271 (KUN)	MN496078	180.9	179.6×	151,704 bp	26,109 bp
*Saxifraga stolonifera*	Cultivation	BGI-SEQ	Folk 258 (FLAS)	MN496079	623.5	238.2×	151,060 bp	25,412 bp
*Sycopsis sinensis*	Cultivation	BGI-SEQ	Folk 256 (FLAS)	MN496080	746.2	117.7×	159,043 bp	26,230 bp

### Phylogenetic analysis

**Fig. 2:**
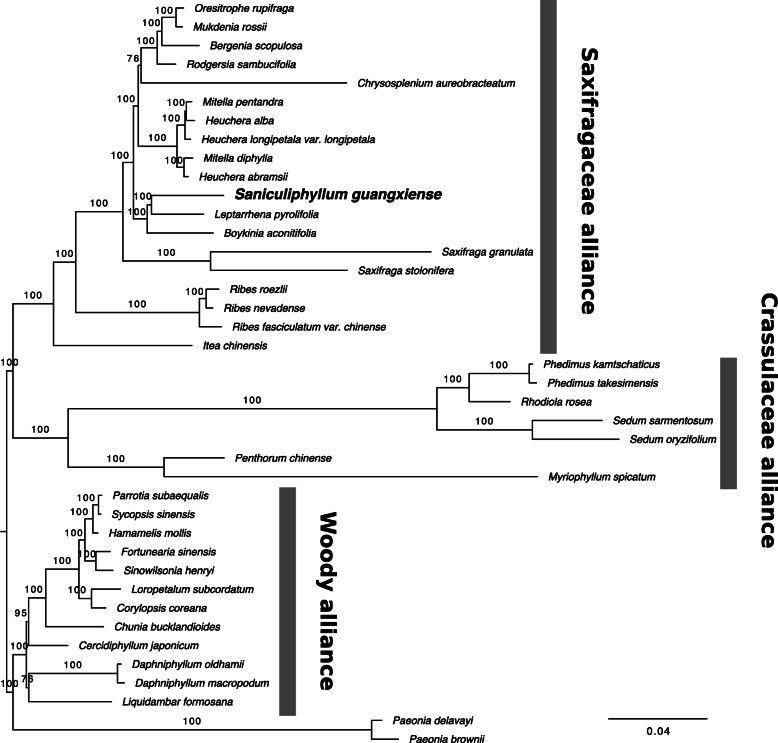
ML phylogeny of Saxifragales plastid genomes. *Saniculiphyllum* shown in bold; labelled clades correspond to the terminology of [26]. The scale bar represents per-site substitution rate. Branch labels represent bootstrap frequencies

The plastome alignment length was 172,773 bp, with 9.9% of the alignment comprising gap characters, and 38,332 parsimony-informative characters excluding the gap characters. Backbone relationships in the chloroplast genome phylogeny were congruent with recent phylogenomic work [18] (Fig. 2). Although receiving maximal bootstrap support, the placement of *Saniculiphyllum* we recovered is different from all previous efforts to place this taxon, none of which agree among themselves and none of which achieved greater than moderate support [16–18]. Our placement resembles previous work [17, 18] in placing *Saniculiphyllum* in a clade comprising the *Astilbe* Buch.-Ham., *Boykinia* Raf.*,* and *Leptarrhena* groups, but the novel placement reported here is sister to *Leptarrhena.*

### Substitution rate analysis

Despite its divergent plastome features, genome-wide substitution rates did not appear elevated in *Saniculiphyllum* on the basis of phylogenetic branch length (Fig. 2), suggesting that at the nucleotide level negative selection is not substantially relaxed in this lineage. We further explored this hypothesis by explicitly testing for the presence of relaxed selection in the seven plastid gene copies with evidence of pseudogenization in *Saniculiphyllum*. We implemented this via *ω* (dN/dS) ratios in PAML [27]. We used a model comparison approach on each gene tree to test whether the *Saniculiphyllum* branch experienced a shift in selection regime compared to its immediately ancestral branch. We did not find evidence of shifts in selection regimes (all *p* > 0.05); likewise, we estimated dN/dS < 1 for the *Saniculiphyllum* branch across all seven pseudogenes (mean 0.0319), consistent with strong negative selection.

## Discussion

### Pseudogenization

In total, we found genomic evidence for seven putative pseudogenes in the *Saniculiphyllum* plastid genome. Five of these (*ndhA, ndhB, ndhD, ndhF*, and *ndhK*), are genes of the *ndh* complex. These genes are highly conserved across the land plants and related green algae [8]. Most losses of plastid gene function have been associated with parasitic and mycoheterotrophic plants, which presumably have few functional constraints on photosynthetic gene evolution. Pseudogenization or loss of genes in the *ndh* complex has nevertheless been observed in several fully photosynthetic lineages with a variety of often highly specialized life history traits: woody perennials in Pinaceae and Gnetales (both gymnosperms), short-lived perennials in Geraniaceae (eudicots: rosids), alpine Circaeasteraceae (eudicots: Ranunculales), carnivorous and often aquatic plants of Lentibulariaceae (eudicots: asterids), various photosynthetic members of Orchidaceae (monocot), and aquatic members of Alismatales (monocot) and Podostemataceae (rosid [1, 3, 5, 8, 10, 13, 28–30];). The primary function of the *ndh* complex is thought to be reduction of photooxidative stress under fluctuating light conditions. While the *ndh* complex appears dispensable under mild growth conditions [9], experimental evidence from knockouts of single *ndh* genes shows that a complete and intact complex is essential for efficient photosynthesis and robust plant growth under stressful conditions [13].

More unusual than loss of *ndh* function is the clear pseudogenization of two other photosynthesis-specific genes, for which we report the first absence in a fully photosynthetic plant. The gene *cemA* encodes a protein involved in carbon uptake; while not essential for photosynthesis, photosynthetic efficiency is reduced under high light environments in *Chlamydomonas* Ehrenb. mutants lacking this gene [31]. The gene *ccsA* encodes a protein involved in heme attachment to chloroplast cytochrome c [32]. *ccsA*, at least in *Chlamydomonas,* is essential for System II photosynthesis [32]. Both *cemA* and *ccsA* are conserved across primary photosynthetic eukaryotes and even cyanobacteria [31, 33].

### Evidence for paralogs in the nucleus

We successfully found and assembled paralogs for all seven novel putative chloroplast pseudogenes in *Saniculiphyllum.* Many of these paralogs are of more conserved sequence than that of the assembled plastid genome; with the exception of *ndhK* these appear to have originated primarily after the divergence of *Saniculiphyllum* from other Saxifragaceae lineages sampled here. On the basis of coverage, these are likely to represent NUPTs (nuclear sequences of plastid origin [34];). Our results are consistent with growing evidence of a slow transfer of organellar gene content into nuclear genomes [14, 34], a process associated with frequent non-homologous recombinational repair between these genomes [35]. While in some cases NUPTs are associated with restoration of function by coding for an imported functional photosynthetic protein [15], it is unlikely that *Saniculiphyllum* has any functional copies of these genes because almost all nuclear paralogs show signs of pseudogenization.

### Other genome anomalies

We also observed unusual CDS terminations upstream or downstream of closely related Saxifragales plastid genomes in four genes (Table S2, Additional file 1); these do not result in frameshifts but expected protein products are of unexpected length. Although less dramatic than the pseudogenization patterns we observed, the lack of length conservation in *Saniculiphyllum* is markedly greater compared to close relatives. Likewise, while the *Saniculiphyllum* plastome is far longer than many non-photosynthetic plants (reviewed in [2]), it is among the shortest in Saxifragales due to large deletions in coding and non-coding regions throughout the plastome.

Despite having one of the most divergent plastid genomes in Saxifragales, there is no evidence for elevated substitution rates in *Saniculiphyllum* based on phylogenetic branch length estimated from the entire plastid genome (Fig. 2). Likewise, we implemented tests on dN/dS ratios in the seven putative pseudogenes, demonstrating that *Saniculiphyllum* does not show significantly different selection regimes at the codon level compared to related lineages. These results suggest that *Saniculiphyllum* primarily differs in its plastid genome evolution via deletions and rare novel stop codons without any detectable global relaxation of purifying selection at the nucleotide level. Dosage of plastid DNA relative to the nucleus also appears to be low in *Saniculiphyllum* compared to relatives*,* likely representing either a reduction in plastids per cell or a reduction in genome copy number per plastid.

### Evolutionary relationships

This work also represents the first robust phylogenomic placement of *Saniculiphyllum*, an important group for interpreting morphological evolution in Saxifragaceae [16]*.* We confirm a close relationship with the *Boykinia* and *Leptarrhena* groups, with which it shares axile placentation, determinate cymose inflorescences, and a strongly rhizomatous habit. However, representatives of the *Astilbe* group and several others have yet to be sampled; denser taxon sampling is needed to confirm the placement reported here.

## Conclusions

Although chloroplast genome evolution in Saxifragales has been previously understood as very conservative [20], further sampling has revealed surprising plastid variation in one of its rarest and most unusual lineages. Similar but less extreme patterns of gene loss have been observed before in aquatic members of order Alismatales and Podostemaceae, and appear to represent multiple independent evolutionary events [3, 5], suggesting a possible relationship with life history. Like these lineages, *Saniculiphyllum* is highly specialized for partially submerged shaded waterfall environments, a stable habitat possibly conducive to relaxed selection on and loss of photosynthetic accessory genes. Nevertheless, this putative correlation is imperfect; while Alismatales contains some of the most thoroughly aquatic-adapted angiosperms, including the only examples of aquatic pollination [3], *Myriophyllum*, a completely aquatic Saxifragales lineage, shows conventional gene content [23], as do many other aquatic plastid genomes (e.g., *Nelumbo* Adans. [36], *Nymphaea* L. [37], *Lemna* L. [38]).

It is tempting to speculate on the relationship between loss of photosynthetic gene content and the imperiled conservation status of *Saniculiphyllum* since loss of abiotic stress-response genes would suggest a poor ability to respond to cope with environmental change. Unfortunately, we understand little of the functional significance of plastid gene content outside of model organisms. We highlight the need for characterization of plastid genome evolution, further examination its relationship to life history traits, and the continued promise of comparative phylogenomic approaches [39] for shedding light on this enigmatic pattern.

## Methods

### Sampling

We sequenced 23 plastomes in total to increase phylogenetic representation. Other than *Saniculiphyllum,* we sampled 16 further taxa of Saxifragaceae to cover most of the major recognized clades recognized in [17], and six further Saxifragales outgroups to increase representation in the woody alliance (cf. [26]). Most materials were obtained from horticultural sources; wild materials were collected with permission from the U.S. National Forest Service. Materials of *Saniculiphyllum*, which unfortunately does not currently have a legal conservation designation, were collected from an unprotected natural area. Sampling localities are given in Table 1. The species sampled in total, listed by major clade names, were: *Darmera* group: *Mukdenia rossii, Oresitrophe rupifraga, Rodgersia sambucifolia; Boykinia* group: *Boykinia aconitifolia; Leptarrhena* group: *Leptarrhena pyrolifolia*; *Heuchera* group: *Heuchera abramsii, Heuchera alba, Heuchera caespitosa, Heuchera eastwoodiae, Heuchera grossulariifolia var. grossulariifolia, Heuchera longipetala* var. *longipetala, Heuchera mexicana* var. *mexicana, Heuchera parvifolia var. utahensis, Mitella diphylla,* and *Mitella pentandra*; *Saxifraga* group: *Saxifraga stolonifera*; incertae sedis: *Saniculiphyllum guangxiense*. Outgroup taxa, listed by family, were: Cercidiphyllaceae: *Cercidiphyllum japonicum.* Daphniphyllaceae: *Daphniphyllum macropodum*; Grossulariaceae: *Ribes nevadense* and *Ribes roezlii*; Hamamelidaceae: *Fortunearia sinensis* and *Sycopsis sinensis*.

### DNA extraction and sequencing

Whole genomic DNAs were isolated from silica-dried leaf material (*Saniculiphyllum*) or fresh material (all other taxa) using a modified CTAB extraction protocol [40]. Although our target was plastid DNA, we sequenced total DNA to enable paralog assembly (see below) and other future related work on mitochondrial and nuclear genomes. Taxa were chosen to represent lineages across Saxifragales. Sequencing was performed either at RAPiD Genomics (Gainesville, Florida, U.S.A.) with 150 bp paired-end Illumina HiSeq sequencing or with 100 bp paired-end BGISEQ-500 sequencing at BGI (Shenzhen, Guangdong, P.R. China), in both cases with an insert size of approximately 300 bp (summarized in Table 1).

### Genome assembly

We used NOVOPlasty v. 3.2 [41] to assemble chloroplast genomes for all sequenced taxa. For each sample, we ran two assemblies using *rbcL* and *matK* seed reference genes from the plastid genome of *Heuchera parviflora* var. *saurensis* R.A. Folk [22]. Reads were not quality filtered following developer recommendations (see https://github.com/ndierckx/NOVOPlasty). We used the following parameters: *k-*mer = 39, expected genome range 120,000–200,000 bp, insert size 300 bp, insert range = 1.8, and insert range strict = 1.3. We also used insert range fine-tuning to account for insert size variation between samples. When running NOVOPlasty on the entire dataset, we found it returned only partial plastid genome assemblies; datasets were normalized to 8 million raw reads per sample for HiSeq data and 4 million for BGI-SEQ samples (~ 100-500X plastid coverage) using standard UNIX tools to achieve full-length assemblies of the plastid genome. Chloroplast genomes in most plants exist in both possible orientations of the small-single copy region relative to the rest of the genome [42], we manually standardized the orientation of the SSC region across samples prior to sequence alignment using Geneious R9.

Annotations were performed in Geneious R9 using the *Heuchera parviflora* reference plastid genome and a cutoff of 70% sequence identity, and draft annotated plastid genomes were aligned and manually examined for annotation accuracy. Additionally, all premature stop codons, inversions, frameshifting indels, and other unusual features were individually verified visually by mapping the original reads back to the assembled plastid genomes using the Geneious read mapping algorithm [43]. We also calculated the percent of chloroplast sequences in the total DNA from these mapped reads using SAMtools [44].

For the seven putative plastid pseudogenes, we searched for potential paralogs in the mitochondrial and nuclear genomes using aTRAM 2 [45, 46]. aTRAM is a method for iterative, targeted assembly that implements commonly used de novo assembly modules on a reduced read set that has sequence homology with a seed sequence. Seed sequences were derived from the CDS sequence of the closest identified relative among our taxa, *Leptarrhena pyrolifolia* (D. Don) Ser. Ten iterations were used per assembly, and the assembler used was SPAdes v. 3.13.0 [47]; other options correspond to defaults. For these analyses, we extracted matching reads from the full *Saniculiphyllum* dataset (~ 180,000,000 reads).

### Phylogenetics

We conducted a maximum likelihood phylogenetic analysis both to reassess the relationships of *Saniculiphyllum* [16–18], and to assess rates of plastid substitution in a phylogenetic context. We analyzed the single-copy plastid sequence from each genome (i.e., with one copy of the inverted repeat), aligned them with MAFFT v. 7.388 [48], and ran phylogenetic analyses in RaxML v. 8.2.10 [49] under a GTR-Γ model with 1000 bootstrap replicates (command “-f a”). Sites were partitioned as either coding (exonic protein-coding, rDNA, and tRNA) or non-coding. For this analysis, we sampled 22 further previously reported plastid genomes (Supplementary Table S1, Additional file 1), as well as generating a plastid genome assembly from previously reported short read data from *Saxifraga granulata* L. ([50]; SRA accession SRX665162), all chosen to represent phylogenetic diversity in Saxifragales, for a total of 40 taxa. We sampled 12 of 16 families, including complete representation of the Saxifragaceae alliance; the plastid of the parasitic family Cynomoriaceae has been sequenced, but this was deliberately excluded as it is on an extremely long branch [51]. Saxifragaceae sampling covers 8 of the 10 major clades recognized in [17]. Tree rooting follows [18].

For the paralog search in aTRAM, we placed recovered sequences in a phylogenetic context by extracting plastid sequences for each gene from the plastid genome alignment, trimming to the extent of chloroplast gene sequences and removing ambiguously aligned regions, and removing any sequences with fewer than 200 bp remaining after these steps. We then built individual gene trees following the RAxML methods above.

### Tests for selection

To test for relaxed selection in putative pseudogenes in *Saniculiphyllum*, we built two models for each of the seven gene trees: a full model allowing *ω* to vary across all branches, and a constrained model where *Saniculiphyllum* was constrained to have the same *ω* as the branch immediately ancestral to it. We used a likelihood ratio test to determine whether the constrained model could be rejected (= a shift in selective regime along this phylogenetic branch). Since multiple tests were executed, multiple comparisons were corrected by the Hochberg method [52].

## Supplementary information

**Additional file 1: Table S1.** Summary of chloroplast genome sequences downloaded from GenBank for phylogenetic analyses. **Table S2.** Summary of premature stop codons, large/frame-shifting indels, and other anomalous genome features unique to *Saniculiphyllum.***Figure S1.** ML gene phylogeny of *ccsA*, showing the phylogenetic placement of *Saniculiphyllum* paralogs (bold) among plastid orthologs. The *Saniculiphyllum* plastid copy is marked ***. Branch labels represent bootstrap frequencies; those below 50 are not plotted. **Figure S2.** ML gene phylogeny of *cemA*, showing the phylogenetic placement of *Saniculiphyllum* paralogs (bold) among plastid orthologs. The *Saniculiphyllum* plastid copy is marked ***. Branch labels represent bootstrap frequencies; those below 50 are not plotted. **Figure S3.** ML gene phylogeny of *ndhA*, showing the phylogenetic placement of *Saniculiphyllum* paralogs (bold) among plastid orthologs. The *Saniculiphyllum* plastid copy is marked ***. Branch labels represent bootstrap frequencies; those below 50 are not plotted. **Figure S4.** ML gene phylogeny of *ndhB*, showing the phylogenetic placement of *Saniculiphyllum* paralogs (bold) among plastid orthologs. The *Saniculiphyllum* plastid copy is marked ***. Branch labels represent bootstrap frequencies; those below 50 are not plotted. **Figure S5.** ML gene phylogeny of *ndhD*, showing the phylogenetic placement of *Saniculiphyllum* paralogs (bold) among plastid orthologs. The *Saniculiphyllum* plastid copy is marked ***. Branch labels represent bootstrap frequencies; those below 50 are not plotted. **Figure S6.** ML gene phylogeny of *ndhF*, showing the phylogenetic placement of *Saniculiphyllum* paralogs (bold) among plastid orthologs. The *Saniculiphyllum* plastid copy is marked ***. Branch labels represent bootstrap frequencies; those below 50 are not plotted. **Figure S7.** ML gene phylogeny of *ndhK*, showing the phylogenetic placement of *Saniculiphyllum* paralogs (bold) among plastid orthologs. The *Saniculiphyllum* plastid copy is marked ***. Branch labels represent bootstrap frequencies; those below 50 are not plotted.

## Data Availability

The datasets supporting the conclusions of this article are available at Dryad (alignments, partition files, and tree topologies; 10.5061/dryad.mgqnk98vt), and at GenBank (accession numbers in Table S1; Additional file 1). Supplemental figures are available in Additional file 1.

## Abbreviations

CDS: Coding sequence
CTAB: Cetrimonium bromide
GTR-Γ: General time reversible with gamma
ITS: Internal transcribed spacer
LSC: Long single copy [region]
NUPT: Nuclear plastid DNA
SSC: Small single copy [region]
SRA: Sequence Read Archive
