# Supplementary material for: Degradation of key photosynthetic genes in the critically endangered semi-aquatic flowering plant Saniculiphyllum guangxiense (Saxifragaceae)
Source: BMC Plant Biol. 2020 Jul 8;20:324. doi: 10.1186/s12870-020-02533-x (PMC7346412; doi:10.1186/s12870-020-02533-x)
Supplement: Supplementary file 1 — Additional file 1: Table S1. Summary of chloroplast genome sequences downloaded from GenBank for phylogenetic analyses. Table S2. Summary of premature stop codons, large/frame-shifting indels, and other anomalous genome features unique to Saniculiphyllum.Figure S1. ML gene phylogeny of ccsA, showing the phylogenetic placement of Saniculiphyllum paralogs (bold) among plastid orthologs. The Saniculiphyllum plastid copy is marked ***. Branch labels represent bootstrap frequencies; those below 50 are not plotted. Figure S2. ML gene phylogeny of cemA, showing the phylogenetic placement of Saniculiphyllum paralogs (bold) among plastid orthologs. The Saniculiphyllum plastid copy is marked ***. Branch labels represent bootstrap frequencies; those below 50 are not plotted. Figure S3. ML gene phylogeny of ndhA, showing the phylogenetic placement of Saniculiphyllum paralogs (bold) among plastid orthologs. The Saniculiphyllum plastid copy is marked ***. Branch labels represent bootstrap frequencies; those below 50 are not plotted. Figure S4. ML gene phylogeny of ndhB, showing the phylogenetic placement of Saniculiphyllum paralogs (bold) among plastid orthologs. The Saniculiphyllum plastid copy is marked ***. Branch labels represent bootstrap frequencies; those below 50 are not plotted. Figure S5. ML gene phylogeny of ndhD, showing the phylogenetic placement of Saniculiphyllum paralogs (bold) among plastid orthologs. The Saniculiphyllum plastid copy is marked ***. Branch labels represent bootstrap frequencies; those below 50 are not plotted. Figure S6. ML gene phylogeny of ndhF, showing the phylogenetic placement of Saniculiphyllum paralogs (bold) among plastid orthologs. The Saniculiphyllum plastid copy is marked ***. Branch labels represent bootstrap frequencies; those below 50 are not plotted. Figure S7. ML gene phylogeny of ndhK, showing the phylogenetic placement of Saniculiphyllum paralogs (bold) among plastid orthologs. The Saniculiphyllum plastid copy is marked ***. Bran [file 12870_2020_2533_MOESM1_ESM.docx]

**Table S1.** Summary of chloroplast genome sequences downloaded from GenBank for phylogenetic analyses.

| **Species** | **Genbank accession** |
| --- | --- |
| *Bergenia scopulosa* | NC_036061 |
| *Cercidiphyllum japonicum* | NC_037940 |
| *Chrysosplenium aureobracteatum* | NC_039740 |
| *Chunia bucklandioides* | NC_041163 |
| *Corylopsis coreana* | NC_040141 |
| *Daphniphyllum oldhamii* | NC_037883 |
| *Fortunearia sinensis* | NC_041487 |
| *Hamamelis mollis* | NC_037881 |
| *Itea chinensis* | MH191391 |
| *Liquidambar formosana* | NC_023092 |
| *Loropetalum subcordatum* | NC_037694 |
| *Mukdenia rossii* | NC_037495 |
| *Myriophyllum spicatum* | NC_037885 |
| *Oresitrophe rupifraga* | NC_037514 |
| *Paeonia brownii* | NC_037880 |
| *Paeonia delavayi* | NC_035718 |
| *Parrotia subaequalis* | NC_037243 |
| *Penthorum chinense* | NC_023086 |
| *Phedimus kamtschaticus* | NC_037946 |
| *Phedimus takesimensis* | NC_026065 |
| *Rhodiola rosea* | NC_041671 |
| *Ribes fasciculatum* var. *chinense* | MH191388 |
| *Saxifraga stolonifera* | NC_037882 |
| *Sedum oryzifolium* | NC_027837 |
| *Sedum sarmentosum* | NC_023085 |
| *Sinowilsonia henryi* | MF497447 |

**Table S2.** Summary of premature stop codons, large/frame-shifting indels, and other anomalous genome features unique to *Saniculiphyllum.*

| **Premature CDS stop codons** | |  | **Unique CDS indels** | | |
| --- | --- | --- | --- | --- | --- |
| **Gene** | **Plastome location** |  | **Gene** | **Length** | **Alignment location** |
| *ndhK*ψ | 51523-51525 |  | *rpoC2* | 9 | 21186-21194 |
| *ndhB*ψ | 96122-96124, 139910-139912 * |  | *rpoC2* | 9 | 21919-21927 |
| *ccsA*ψ | 112746-112747 |  | *rpoC2* | 3 | 22736-22738 |
| *ccsA*ψ | 112755-112757 |  | *psaA* | 15 | 48026-48040 |
| *ccsA*ψ | 112806-112808 |  | *atpB* | 5 | 63018-63023 |
| *ccsA*ψ | 112827-112829 |  | *accD* | 12 | 67580-67592 |
| *ccsA*ψ | 112833-112835 |  | *accD* | 12 | 67698-67709 |
| *ccsA*ψ | 112860-112862 |  | *accD* | 12 | 68112-68123 |
| *ccsA*ψ | 112863-112865 |  | *cemA*ψ | 163 | 72766-72928 |
| *ccsA*ψ | 112872-122874 |  | *rpoA* | 6 | 91177-91183 |
| *ccsA*ψ | 112878-112880 |  | *rpl22* | 62 | 96810-96871 |
| *ccsA*ψ | 112926-112928 |  | *ycf1*ψ | 24 | 124096-124119 |
| *ccsA*ψ | 112935-112937 |  | *ycf1*ψ | 204 | 124803-125006 |
| *ccsA*ψ | 112959-112961 |  | *ndhF*ψ | >330 | 126925-127254 |
| *ccsA*ψ | 112989-112991 |  | *ccsA*ψ | 4 | 130634-130637 |
| *ccsA*ψ | 113025-113027 |  | *ndhD*ψ | 126 | 131929-132054 |
| *ccsA*ψ | 113094-113096 |  | *ndhA*ψ | 1 | 136337 |
| *ccsA*ψ | 113136-113138 |  | *ycf1* | 12 | 140640-140651 |
| *ccsA*ψ | 113151-113153 |  | *ycf1* | 8 | 141363-141368 |
| *ccsA*ψ | 113157-113159 |  | *ycf1* | 30 | 143910-143939 |
| *ccsA*ψ | 113337-113339 |  | *ycf1* | 24 | 145783-145806 |
| *ccsA*ψ | 113370-113372 |  |  |  |  |
| *ccsA*ψ | 113376-113378 |  | **Miscellaneous anomalous CDS features** | | |
| *ndhD*ψ | 113742-113744 |  | **Gene** | **Type** | **Plastome location** |
| *ndhD*ψ | 113745-113747 |  | *ndhK*ψ | Inversion | 51518-51524 |
| *ndhD*ψ | 113877-113879 |  | *atpB* | Unconventional CDS termination | 3 bp upstream |
| *ndhD*ψ | 113883-113885 |  | *cemA*ψ | Unconventional CDS termination | 15 bp downstream |
| *ndhD*ψ | 113898-113900 |  | *rpl20* | Unconventional CDS termination | 21 bp downstream |
| *ndhD*ψ | 113910-113912 |  | *ycf2* | Unconventional CDS termination | 15 bp upstream |
| *ndhD*ψ | 113913-113915 |  | *ndhA*ψ | Expected stop codon missing | 117750-117752 |
| *ndhD*ψ | 113934-113936 |  |  |  |  |
| *ndhD*ψ | 114030-114032 |  |  |  |  |
| *ndhD*ψ | 114066-114068 |  |  |  |  |
| *ndhD*ψ | 114087-114089 |  |  |  |  |
| ndhDψ | 114120-114122 |  |  | |  |
| *ndhD*ψ | 114138-114140 |  |  | |  |
| *ndhD*ψ | 114432-112434 |  |  | |  |
| *ndhD*ψ | 114444-114446 |  |  |  |  |
| *ndhD*ψ | 114462-114464 |  |  |  |  |
| *ndhA*ψ | 117792-117790 |  |  |  |  |
| *ndhA*ψ | 117853-117855 |  |  |  |  |
| *ndhA*ψ | 117904-117906 |  |  |  |  |
| *ndhA*ψ | 117955-117957 |  |  |  |  |
| *ndhA*ψ | 117964-117966 |  |  |  |  |
| *ndhA*ψ | 117973-117975 |  |  |  |  |

Notes: * Two copies, one in each IR region. ψ Putative pseudogene. > Indel extends beyond gene. Note for *ycf1*: as with many other chloroplast genomes, both a functional and pseudogenized copy exist for this gene.

**Figure S1.** ML gene phylogeny of *ccsA*, showing the phylogenetic placement of *Saniculiphyllum* paralogs (bold) among plastid orthologs. The *Saniculiphyllum* plastid copy is marked ***. Branch labels represent bootstrap frequencies; those below 50 are not plotted.

**Figure S2.** ML gene phylogeny of *cemA*, showing the phylogenetic placement of *Saniculiphyllum* paralogs (bold) among plastid orthologs. The *Saniculiphyllum* plastid copy is marked ***. Branch labels represent bootstrap frequencies; those below 50 are not plotted.

**Figure S3.** ML gene phylogeny of *ndhA*, showing the phylogenetic placement of *Saniculiphyllum* paralogs (bold) among plastid orthologs. The *Saniculiphyllum* plastid copy is marked ***. Branch labels represent bootstrap frequencies; those below 50 are not plotted.

**Figure S4.** ML gene phylogeny of *ndhB*, showing the phylogenetic placement of *Saniculiphyllum* paralogs (bold) among plastid orthologs. The *Saniculiphyllum* plastid copy is marked ***. Branch labels represent bootstrap frequencies; those below 50 are not plotted.

**Figure S5.** ML gene phylogeny of *ndhD*, showing the phylogenetic placement of *Saniculiphyllum* paralogs (bold) among plastid orthologs. The *Saniculiphyllum* plastid copy is marked ***. Branch labels represent bootstrap frequencies; those below 50 are not plotted.

**Figure S6.** ML gene phylogeny of *ndhF*, showing the phylogenetic placement of *Saniculiphyllum* paralogs (bold) among plastid orthologs. The *Saniculiphyllum* plastid copy is marked ***. Branch labels represent bootstrap frequencies; those below 50 are not plotted.

**Figure S7.** ML gene phylogeny of *ndhK*, showing the phylogenetic placement of *Saniculiphyllum* paralogs (bold) among plastid orthologs. The *Saniculiphyllum* plastid copy is marked ***. Branch labels represent bootstrap frequencies; those below 50 are not plotted.
